# Individual differences in dissonance arousal/reduction relate to physical exercise: Testing the action-based model

**DOI:** 10.1371/journal.pone.0275990

**Published:** 2022-10-13

**Authors:** Eddie Harmon-Jones, Cindy Harmon-Jones

**Affiliations:** School of Psychology, The University of New South Wales, Sydney, New South Wales, Australia; Polytechnic Institute of Coimbra: Instituto Politecnico de Coimbra, PORTUGAL

## Abstract

**Introduction:**

The present research was designed to test predictions derived from the action-based model of cognitive dissonance theory. These predictions were that dissonance arousal would be negatively related to effective behavior, and that dissonance reduction would be positively related to effective behavior.

**Method:**

Dissonance arousal and reduction were measured using an individual differences questionnaire. Effective behavior was measured as amount of physical exercise obtained from an exercise app that measures exercise using GPS (cycling kilometers over one year; Study 1) and from self-reports (number of days during the previous week; Study 2–3).

**Results:**

Results suggested that individual differences in dissonance arousal relate to less exercise and that individual differences in dissonance reduction relate to more exercise. Statistically controlling for trait approach and avoidance motivation as well as satisfaction with life revealed that dissonance processes predicted exercise behavior over these traits. This pattern of results was generally consistent across the three studies. Moreover, results from Studies 2–3 suggested possible statistical mediators from the exercise commitment literature of the relationship between trait dissonance arousal/reduction and exercise behavior.

**Discussion and conclusion:**

These results highlight the importance of considering dissonance processes as adaptive ones, and they suggest possible ways of increasing exercise behavior.

## Introduction

We designed the present research to test the prediction derived from the action-based model [1, 2] that dissonance arousal would be negatively associated with effective behavior and that dissonance reduction would be positively associated with effective behavior. In the context of physical exercise, we posited that effective behavior could be operationalized as exercising more often. Understanding which psychological variables relate to exercising may aid in increasing exercise, which improves cognitive functions, mood, and mental health [3, 4]. For example, engaging in regular physical activity several times a week reduces depression [5], slows cognitive aging [6], and reduces stress responses [7]. Moreover, greater frequency and duration of exercise is related to lower depression [8].

Cognitive dissonance theory [9] posits that dissonance (psychological discomfort) is aroused when organisms are confronted with conflicting cognitions. Dissonance arousal may then motivate organisms to engage in cognitive or behavioral strategies to reduce the psychological discomfort, and this “dissonance reduction” generally involves decreasing the conflict between cognitions [10]. Dissonance theory has been tested in a variety of ways. Three of most common of these are the effort justification paradigm, difficult decision paradigm, and induced compliance paradigm [11]. In the effort justification paradigm, dissonance occurs because exerting unpleasant effort is inconsistent with one’s desire of not engaging in such effort. Individuals often reduce dissonance regarding effort by valuing the reason for engaging in the effort [12, 13]. After a difficult decision, dissonance occurs because the positive aspects of the rejected decision option and the negative aspects of the chosen decision option are inconsistent with the decision. Individuals often reduce this dissonance by increasing their value of the chosen option and decreasing their value of the rejected option as compared to the ratings of these options prior to the decision [14, 15]. When individuals are subtly induced (not forced) to comply with a request to behave in ways counter to their attitudes, dissonance occurs because their original attitude is inconsistent with their behavior. They often reduce this dissonance by changing their attitudes to be more consistent with their recent behavior [16, 17]. Other research paradigms also exist, but these are the main paradigms and ways of reducing dissonance employed in research on cognitive dissonance.

Each of these dissonance paradigms or situations can be considered a commitment situation [18]. Individuals commit to a chosen course of action, to engage in unpleasant effort, decide between options, or enact a counter attitudinal behavior. This commitment then guides dissonance reduction, such that the commitment is often supported and enhanced by the cognitive or behavioral changes [11, 18].

Building on this insight, one extension of the original theory, the action-based model [2, 19], posits that organisms experience dissonance because conflicting cognitions have the potential to interfere with behaving effectively. That is, cognitions that conflict with one another have conflicting action tendencies. Organisms then reduce the discrepancy between cognitions in order to behave effectively; they cognitively work to support and enhance the chosen commitment. As such, dissonance arousal should be associated with less effective behavior, and dissonance reduction should be associated with more effective behavior.

These ideas have not been directly tested in dissonance research, but other research provides evidence consistent with these predictions. For instance, Shah et al. [20] found individuals committed to a goal were more likely to have that goal highly accessible and have alternative goals cognitively inhibited; moreover, both goal accessibility and inhibition of alternative goals predicted better task performance. In dissonance theory terms, these committed individuals were more likely to focus on consonant cognitions (committed goal) and ignore dissonant cognitions (alternative goal), and these cognitive processes were associated with more effective behavior (task performance). In addition, within committed romantic relationships, attention to attractive alternatives predicts relationship failure [21]. In this example, attention to dissonant cognitions (the attractive alternative) predicts less effective behavior with regard to the romantic relationship.

The majority of past dissonance studies have tested predictions derived from the theory with experimental manipulations. However, some research suggests that individual differences in dissonance arousal and dissonance reduction can be measured, and these individual differences relate to other important variables [22]. The Dissonance Arousal and Reduction Questionnaire (DARQ) measures these individual differences [22]. As revealed in confirmatory factor analyses, the DARQ is comprised of 6 lower-order factors (3 dissonance situations X arousal/reduction) and 2 higher-order factors (arousal vs. reduction). These individual difference measures of dissonance arousal and dissonance reduction correlate negatively with each other [22]. Moreover, dissonance arousal correlates positively with Personal Fear of Invalidity and Response to Lack of Structure [23, 24]. These latter scales measure individual differences in preference for cognitive simplicity and structure. In contrast, dissonance reduction correlates positively with Desire for Structure and negatively with Fear of Invalidity. These correlations provide convergent validity of the DARQ, but because the correlations were of moderate size, these correlations also suggest that the DARQ measures different constructs than these other scales.

Moreover, dissonance arousal correlates negatively with subjective wellbeing (e.g., Satisfaction with Life Scale [SWLS]; [25]), whereas dissonance reduction correlates positively with the SWLS [22]. In addition, dissonance arousal correlates positively with avoidance motivation as measured by the Behavioral Inhibition Scale (BIS), whereas dissonance reduction correlates positively with approach motivation as measured by the Behavioral Activation Scale (BAS; of the BIS/BAS scales from [26]). Because BIS/BAS and SWLS relate to DARQ and to physical exercise, we measured them in the current research to assess how they also related to physical exercise. Based on past research [27–30], BAS and SWLS should be related to more physical exercise, and BIS should be related to less physical exercise. In addition, we tested whether DARQ predicted physical exercise when statistically controlling for BIS/BAS and SWLS. Thus, these analyses would test whether dissonance-related variables (as measured by DARQ) predict physical exercise when statistically controlled for third variables that have been found to be associated with both DARQ and physical exercise in previous research [22, 27–30].

Physical exercise may be considered a measure of the construct effective behavior for the following reason. If an individual has a goal to engage in physical exercise, then they would be behaving effectively if they engaged in more physical exercise. Individuals have decided to engage in exercise and then they effectively enact that decision by engaging in more physical exercise. On the way to effectively engaging in exercise, individuals may cognitively reduce the dissonance associated with making the decision to engage in exercise (which may be effortful) by: 1) feeling more satisfied with the exercise (adding consonant cognitions or increasing the importance of them); 2) shielding themselves from attractive alternative options (decreasing dissonant cognitions); 3) feeling more motivated to, and personally invested in, the exercise; 4) and perceiving more social support for their exercise.

In Study 1, we tested this hypothesis by examining the relationships of individual differences in dissonance arousal and dissonance reduction with physical exercise, in individuals who have chosen to exercise. To measure physical exercise, we recruited individual who use Strava, an online platform that records GPS data (distance, time) for cycling, running, and swimming. From Strava, we collected the number of kilometers and the number of minutes each participant exercised in one year.

## Study 1

### Method

#### Participants

Participants were recruited through email and social media (e.g., Facebook, Instagram, Strava clubs, Slow Twitch). The recruitment message invited persons to complete the survey if they had a Strava account and recorded at least one run, swim, or cycle per week. Individuals were also asked to share the message with others who might have Strava accounts and could complete the survey. We did not conduct a priori power analyses. We simply collected as many responses as we could in the allotted timeframe for this study. Data analyses did not commence until all data were collected. Because of a reviewer’s request, we conducted a sensitivity power analysis from G*Power 3.1.9.7 [31], to compute the effect size (for our already obtained sample) that would be required for 80% power to find a significant effect when one truly exists and a 5% or less chance for the observed results given that the null hypothesis is true (one-tailed). With 93 participants (the number in this study), the critical r is .25.

Participants were 93 individuals (25 female, 68 male) who had an accessible Strava account for all of 2020 (verified by checking if they used Strava in 2019) and completed the survey (conducted during 2020). Participants were from various countries, predominantly Australia, England, and the USA. Participants were of the following ethnicities: 81 European/White, 1 African/Black, 7 Asian, 4 other. Participants were between 18 and 72 years of age (*M* = 40.34, *SD* = 13.61). Participants were not remunerated for their time. The UNSW Human Research Ethics Advisory Panel for Psychology approved this study as well as the following studies (file 3068). Australian research with humans is conducted under the National Statement on Ethical Conduct in Human Research, which is based on the Declaration of Helsinki. Data collection and analyses complied with the terms and conditions of the source of the data in this and all other studies in this article.

#### Procedure

In the Qualtrics online survey, participants first read a consent form and then indicated that they consented to participate by checking an agreement box. All studies in this article used this type of consent procedure. That is, written informed consent was obtained from all participants for inclusion in the study. Then, they were prompted to provide their Strava ID before giving their demographic information (age, gender, and ethnicity). Then, participants completed the following questionnaires.

The Dissonance Arousal and Reduction Questionnaire (DARQ; [22]) consists of 23 statements with which participants express their agreement on a 6-point scale (strongly disagree, moderately disagree, slightly disagree, slightly agree, moderately agree, strongly agree). The questionnaire contains two higher-order factors (questionnaires): dissonance-arousal-overall (Cronbach’s alpha = .86) and dissonance-reduction-overall (Cronbach’s alpha = .76). Each of these higher-order factors are comprised of three sub-factors: effort-dissonance-arousal (3 items; e.g., “After I work hard on something, I often wish I hadn’t bothered”; Cronbach’s alpha = .71); effort-dissonance-reduction (5 items; e.g., “My favorite things are the things I’ve had to work the hardest to get”; Cronbach’s alpha = .80); decision-dissonance-arousal (4 items; e.g., “I often regret my decisions”; Cronbach’s alpha = .78); decision-dissonance-reduction (4 items; e.g., “Typically, I appreciate what I decided to do”; Cronbach’s alpha = .56); induced-compliance-dissonance-arousal (3 items; e.g., “I feel really bad about myself if I do something stupid”; Cronbach’s alpha = .78); and induced-compliance-dissonance-reduction (4 items; “I can think of good reasons for things I’ve done, even things that might seem foolish to someone else”; Cronbach’s alpha = .63).

The Behavioral Inhibition System (BIS) and Behavioral Activation System (BAS) scales [26] contains 20 statements with which participants indicate how well each describes themselves (very false for me, somewhat false for me, somewhat true for me, very true for me). BIS sensitivity assesses concerns about possible or actual negative occurrences (7 items; e.g., "I worry about making mistakes"; Cronbach’s alpha = .82). BAS sensitivity assesses the driven pursuit of appetitive goals (4 items; "I go out of my way to get things I want"; BAS-Drive; Cronbach’s alpha = .86), responsiveness to reward (5 items; "When I get something I want I feel excited and energized"; BAS-Reward Responsiveness; Cronbach’s alpha = .75), and a tendency to seek new rewards (4 items; "I’m always willing to try something new if I think it will be fun"; BAS-Fun Seeking; Cronbach’s alpha = .69).

The Satisfaction with Life Scale (SWLS; [25]) contains five statements with which participants express their agreement (strongly disagree, disagree, slightly disagree, neither agree nor disagree, slightly agree, agree, strongly agree). An example statement is, “In most ways my life is close to my ideal” (Cronbach’s alpha = .89).

To analyze the results, we used SPSS version 27 and Statistica version 14. Following the common convention, we set alpha at .05 (two-tailed), but considered alphas less than .10 as significant for directional hypothesis tests (because this would be .05 for a directional test).

### Results

Participants spent more time cycling than running, and they spent the least amount of time swimming. A repeated measures ANOVA revealed a significant main effect of exercise type, *F* (2, 181) = 43.40, *p* < .001, *partial eta squared* = 0.32. Follow-up LSD tests revealed that each exercise differed from the other two (*ps* < .001). The ordering of time (minutes) spent was as follows: cycling (*M* = 10,018.29; *SD* = 10,445.65); running (*M* = 4,568.61; *SD* = 5,356.16); swimming (*M* = 410.76; *SD* = 1,788.42). In addition to these differences in time, 16 participants did not cycle at all during 2020, 17 did not run at all; 67 did not swim at all (no participants had 0s for all three exercise types). Because of these differences between types of exercise, we analyzed exercise type separately.

Bivariate correlations are shown in Table 1. Dissonance arousal and dissonance reduction correlated with some cycling- and swimming-related variables in the predicted directions. That is, dissonance arousal was negatively correlated with cycling and swimming, whereas dissonance reduction was positively correlated with cycling and swimming. Running, however, was not correlated with either dissonance arousal or dissonance reduction.

**Table 1:** Correlations of dissonance-related variables with exercise variables (Study 1).

	Cyc km	Cyc min	Run km	Run min	Swim km	Swim min
**Ds Aro**	-.24**	-.26**	.08	.05	-.15	-.15
**Eff Aro**	-.08	-.10	.07	.05	-.05	-.05
**Dec Aro**	-.28***	-.30***	.10	.09	-.13	-.13
**Ind Aro**	-.22**	-.23**	.04	-.01	-.16	-.17
**Ds Red**	.20*	.18*	-.02	-.03	.18*	.17
**Eff Red**	.12	.11	.02	.03	.03	.03
**Dec Red**	.25**	.26**	-.18	-.17	.16	.15
**Ind Red**	.07	.04	.09	.08	.19*	.19*
**Age**	.17	.23**	-.04	.01	.11	.12
**Gender**	-.13	-.17	.01	.02	-.05	-.06

*Note*.

* is *p* < .10

** is *p* < .05

*** is *p* < .01 (two-tailed tests; because some predictions were directional, we used the *p* < .10 as a cutoff value, which would be *p* < .05 one-tailed). Ds Arou = dissonance arousal overall; Eff Arou = effort justification arousal; Dec Arou = decision arousal; Ind Arou = induced compliance arousal; Ds Redu = dissonance reduction overall; Eff Redu = effort justification reduction; Dec Redu = decision reduction; Ind Redu = induced compliance reduction.

Because age and gender were significantly correlated with some of the key variables of interest (e.g., cycling km, cycling min, dissonance arousal), the primary predictions were tested again with multiple regression analyses in which the outcome variable (e.g., cycling km) was predicted (separately) by each dissonance-related variable, age, and gender. These regressions were conducted so that the effects of age and gender could be statistically controlled. As shown in Table 2, these dissonance variables continued to correlate in the same ways with exercise variables; these results suggest that age and gender do not explain the relationship between dissonance variables and exercise.

**Table 2:** Partial correlations of cycling, running, and swimming variables with dissonance variables controlling for gender and age (Study 1).

	Cyc km	Cyc min	Run km	Run min	Swim km	Swim min
**Dis Aro**	-.18*	-.18*	.07	.05	-.11	-.11
**Eff Aro**	-.04	-.06	.07	.06	-.03	-.02
**Dec Aro**	-.23**	-.23**	.09	.10	-.09	-.09
**Ind Aro**	-.16	-.15	.02	-.00	-.13	-.13
**Dis Red**	.20*	.18*	-.02	-.02	.18*	.17
**Eff Red**	.14	.13	.02	.03	.04	.04
**Dec Red**	.23**	.23**	-.17	-.18	.14	.14
**Ind Red**	.07	.05	.09	.08	.20*	.19*

*Note*.

* *p* < .10

** *p* < .05

***p < .01. Ds Aro = dissonance arousal overall; Eff Aro = effort justification arousal; Dec Aro = decision arousal; Ind Aro = induced compliance arousal; Ds Red = dissonance reduction overall; Eff Red = effort justification reduction; Dec Red = decision reduction; Ind Red = induced compliance reduction.

Next, we examined the correlations of dissonance variables with other individual differences variables, age, and gender. As shown in Table 3, dissonance arousal variables were positively correlated with BIS but negatively correlated with satisfaction with life and age. In addition, some of the dissonance arousal variables were higher in women than in men. In contrast, dissonance reduction variables were positively correlated with some BAS variables, but not significantly correlated with satisfaction with life (except for decision reduction), age, or gender. These results of dissonance variables being correlated with BIS, BAS, and satisfaction with life are consistent with past research [22].

**Table 3:** Correlations of dissonance variables with BIS, BAS, SWLS, age, and gender (Study 1).

	BIS	BAS D	BAS FS	BAS RR	SWLS	Age	Gender
**Ds Aro**	.64***	.01	.16	.23**	-.31***	-.39***	.22**
**Eff Aro**	.39***	-.04	.07	.06	-.25**	-.20*	.09
**Dec Aro**	.54***	.03	.16	.14	-.34***	-.31***	.31***
**Ind Aro**	.63***	.02	.15	.32***	-.22**	-.41***	.16
**Ds Red**	-.12	.41***	.08	.30***	.14	-.00	-.05
**Eff Red**	.14	.46***	.20*	.35***	.05	-.10	-.01
**Dec Red**	-.31***	.24**	.07	.22**	.35***	.16	-.10
**Ind Red**	-.10	.19*	-.10	.09	-.07	-.05	-.01

*Note*.

* *p* < .10

** *p* < .05

***p < .01.

Ds Aro = dissonance arousal overall; Eff Aro = effort justification arousal; Dec Aro = decision arousal; Ind Aro = induced compliance arousal; Ds Red = dissonance reduction overall; Eff Red = effort justification reduction; Dec Red = decision reduction; Ind Red = induced compliance reduction. BIS = Behavioral Inhibition Scale; BAS = Behavioral Activation Scale total score (FS = fun-seeking; RR = reward responsiveness); SWLS = Satisfaction with Life Scale.

Next, we examined the correlations of BIS, BAS, and satisfaction with life with exercise variables (see Table 4). For cycling, number of minutes was negatively correlated with BIS; that is, individuals who scored higher in BIS cycled fewer minutes. Also, satisfaction with life was positively correlated with cycling kms and min; that is, individuals who scored higher in BAS cycled more. Swimming also showed some significant correlations but because swimming was not correlated with dissonance variables, they are not discussed.

**Table 4:** Correlations of BIS/BAS and SWLS with exercise variables (controlling for age and gender) (Study 1).

	Cyc km	Cyc min	Run km	Run min	Swim km	Swim min
**BIS**	-.08	-.09	.12	.07	.05	.06
**BAS Drive**	.19*	.15	.01	.04	.20*	.20*
**BAS FS**	-.08	-.08	.13	.15	-.21**	-.21**
**BAS RR**	.23**	.20*	.05	.02	.22**	.22**
**SWLS**	.22**	.24**	-.17	-.15	.02	.01

*Note*.

* *p* < .10

** *p* < .05

***p < .01.

BIS = Behavioral Inhibition Scale; BAS = Behavioral Activation Scale total score (FS = fun-seeking; RR = reward responsiveness); SWLS = Satisfaction with Life Scale. Note that SWLS is negatively correlated with running km and min; this is opposite to research that has found SWLS relates positively to exercise.

Because SWLS was significantly correlated with the cycling variables, we conducted multiple regressions in which dissonance-related variables (that were significantly correlated with cycling variables) and SWLS were used as simultaneous predictors of cycling, to test whether dissonance variables related to cycling because of SWLS. As shown in Table 5, the results do not support the idea that dissonance-related variables related to cycling because of SWLS. That is, when predicting cycling in multiple regressions, both SWLS and dissonance-related variables were independent predictors. If SWLS accounted for the effects of dissonance-related variables on cycling, then SWLS should have been a highly significant predictor of cycling and dissonance-related variables should have been non-significant predictors. However, the results are not consistent with this.

**Table 5:** Partial correlations of cycling variables with dissonance variables and SWLS and BIS/BAS (Study 1).

	Cyc km	Cyc min
**Ds Aro**	-.19*	-.19*
**BIS**	.00	-.02
**Ds Red**	.18*	.16
**BIS**	-.13	-.16
**Ds Aro**	-.25**	-.27***
**BAS**	.11	.06
**Ds Red**	.19*	.19*
**BAS**	.00	-.05
**Ds Aro**	-.18*	-.20*
**SWLS**	.16	.18*
**Ds Red**	.19*	.19*
**SWLS**	.15	.17*

*Note*.

* *p* < .10

** *p* < .05

***p < .01.

Cyc km = Cycling km in one year; Cyc min = number of min cycled in one year; Ds Aro = dissonance arousal overall; Eff Aro = effort justification arousal; Dec Aro = decision arousal; Ind Aro = induced compliance arousal; Ds Red = dissonance reduction overall; Eff Red = effort justification reduction; Dec Red = decision reduction; Ind Red = induced compliance reduction. BIS = Behavioral Inhibition Scale; BAS = Behavioral Activation Scale total score; SWLS = Satisfaction with Life Scale. In these regressions, one dissonance-related variable (e.g., Ds Aro) and one alternative variable (e.g., BIS) were simultaneously entered to predict one physical activity variable (e.g., Cyc km).

### Discussion

The results of Study 1 supported the hypothesis that individual differences in dissonance arousal would be negatively correlated with amount of exercise and that individual differences in dissonance reduction would be positively correlated with amount of exercise. However, support for the hypothesis occurred primarily with cycling, somewhat with swimming, and not at all with running. It is difficult to know why these differences occurred as a function of exercise type. However, it is important to note that only cycling was correlated with SWLS in a manner consistent with past research (more SWLS was correlated with more cycling).

Perhaps the participants in our sample who were primarily runners or swimmers differed in important ways from those who were primarily cyclists. To try to understand this, we created three groups of participants based on their primary exercise. No participants were primarily swimmers as measured by their relative amount of each exercise (using the triathlon ratio of 15:4:1 for swim, run, cycle). Participants were coded as cyclists if their cycling kms were at least 4 times greater than running (*n* = 39); participants were coded as runners if their running kms were less than 4 times their cycling kms (*n* = 36); and other participants were coded as a combined group of duathletes and triathletes (*n* = 18). To understand why runners differed from cyclists, these two groups were compared on age and gender. The cyclists in the sample were older (*M* = 43.21, *SD* = 13.49) than the runners (*M* = 36.69, *SD* = 13.58), *t* (73) = 2.08, *p* = .034. In addition, the cyclists group contained more men (*n* = 33) than women (*n* = 6), whereas the runners group contained approximately equal numbers of men (*n* = 20) and women (*n* = 16). A Mann-Whitney U Test revealed a significant effect of group, *Z adjusted* = 2.74, *p* = .006. These differences between cyclists and runners may have contributed to the differences in correlations. However, controlling for gender and age did not significantly influence the correlations with cycling. We also tested the correlations for only men vs. only women. The men revealed a similar pattern of results to the full sample, and the women revealed no significant correlations. However, it should be noted that there were 68 men and only 25 women.

Another way to examine these data is to test the correlation of running kms (and min) with dissonance arousal and reduction (only the full scale dissonance arousal and reduction variables were used) within each of the three groups identified earlier. All three groups of participants ran at least somewhat. These tests would assist in determining whether the activity of running or the group who were primarily runners were the outliers in terms of dissonance predicted correlations. Within the cyclists group, dissonance reduction correlated positively with running kms (*r* = .33, *p* = .04) and min (*r* = .33, *p* = .04), but dissonance arousal did not correlated significantly with either kms (*r* = .08, *p* = .65) or min (*r* = .09, *p* = .57). Within the runners group, no significant correlations occurred for dissonance reduction (with kms, *r* = -.001, *p* = .995; with min, *r* = .001, *p* = .994). However, dissonance arousal correlated negatively but non-significantly with running kms (*r* = -.26, *p* = .12) and min (*r* = -.31, *p* = .06); these correlations are in the predicted direction. Within the duathlete group, no correlations were significant, but the ones with dissonance reduction were in the prediction direction (with kms, *r* = .42, *p* = .08; with min, *r* = .45, *p* = .06), whereas the ones with dissonance arousal were not (with kms, *r* = .13, *p* = .60; with min, *r* = .12, *p* = .63). These results suggest that the group who were primarily runners may have been unusual in some way.

The effect of primary exercise group on dissonance arousal and dissonance reduction was tested in a 2 (group: cyclists vs. runners) X 2 (scale) mixed ANOVA. It revealed a significant interaction, *F*(1, 73) = 12.32, *p* < .001. Follow-up LSD tests revealed that both groups scored higher in dissonance reduction than dissonance arousal (*ps* < .001). However, the cyclist group scored lower (*M* = 2.44, *SD* = .74) than the runner group (*M* = 3.08, *SD* = .87) on dissonance arousal (*p* < .001), but the two groups did not differ significantly on dissonance reduction (*p* = .16). Although these differences existed, it is not clear how they would explain the lack of significant correlations with exercise distance or time in the runners group.

## Study 2

Because Study 1 provided some evidence suggestive of a relationship between dissonance-related variables and exercise, we conducted a second study to test whether dissonance-related variables would relate to exercise. To measure exercise in Study 2, we asked participants to report on the amount they had exercised in the previous week. We did this because of the difficulties we experienced with recruiting participants in Study 1. Prior to conducting Study 1, we attempted to collect a large sample via crowdsourcing (Mturk) and required participants to have a Strava ID. However, we quickly learned that many participants would simply sign up for Strava just prior to completing the Mturk survey. Consequently, we changed participant recruitment for Study 1, as noted above. Recruitment took several months, and we probably exhausted those sources of participants. Thus, in Study 2, rather than use Strava, we used self-reports to measure exercise.

In addition to re-examining the variables from Study 1 in Study 2, we tested some new hypotheses in Study 2. Research on the psychological determinants of exercise behavior has suggested that commitment to exercise plays an important role in influencing exercise [32]. Along these lines, the sport commitment model proposes that commitment, or “the desire and resolve to continue sport participation”, is influenced by a number of variables [33, p. 6]. Based largely on this model, the Exercise Commitment Scale (ECS) was developed [34]. Research on this scale extended the exercise commitment model by proposing that commitment should be split into two types of commitment: commitment because of external control or obligation (“have to”) and commitment because choice and personal desire (“wanting to”). This split was based on motivational research by Ryan and Deci [35]. Thus, the scale contains items designed to measure “want to commit” vs. “have to commit”. In addition to these two dimensions of commitment, the scale contains subscales that measure determinants of commitment. These are: satisfaction, personal investment, social constraints (norms or expectations that induce felt obligation to continue exercising), social support, and involvement alternatives. Research using this scale has found that exercise behavior is correlated positively with want-to-commit, satisfaction, personal investment, and social support [34].

When considering this exercise commitment model and scale in relation to dissonance theory and more specifically individual differences in dissonance arousal and reduction, we posited that the broader, more distal personality characteristics of dissonance arousal/reduction might influence the commitment to exercise, which would then influence the amount of exercise. That is, individuals who score higher in dissonance arousal may be lower in want-to commitment to exercise. They may also have less satisfaction with exercise and less personal investment, but perceive more involvement alternatives to exercise. On the other hand, individuals who score higher in dissonance reduction may be higher in want-to commitment. They may also have more satisfaction with exercise and more personal investment, and perceive fewer involvement alternatives to exercise. The variables from the exercise commitment model are more proximal to exercise behavior and thus may serve as mediators of the relationship between the more distal dissonance variables and exercise. We tested these mediational predictions in Study 2 after presenting the results that would conceptually replicate the results of Study 1.

### Method

#### Participants

Recruited from Amazon’s Mechanical Turk (Mturk) to complete a study on physical exercise and personality, participants were 282 individuals (125 female, 157 male) who lived in the USA. Participants were of the following ethnicities: 193 European/White, 38 African/Black, 24 Asian, 21 Hispanic/Latino, 2 Native American, and 6 other. Participants were between 18 and 77 years of age (*M* = 39.36, *SD* = 12.03). An additional 22 respondents completed the survey but their data was not analyzed because they did not correctly answer attention check questions embedded in the questionnaires (e.g., After reading this, select “moderately agree” for your response) or because they did not give sensible answers to open-ended questions (e.g., Tell me about the kind(s) of exercise you did when you were of high school age.). Participants were paid $1.50 US for their time.

An a priori power analysis using G*Power 3.1.9.7 [35] indicated a sample of 258 would be needed for *r* = .20, *p* < .05, 90% power. Therefore, we aimed to collect responses from approximately 300 individuals, knowing that some responses may be unusable for failed attention checks etc.

#### Procedure

After participants provided informed consent and demographics (age, gender, ethnicity), they were asked to indicate their primary exercise, whether they had a specific reason for training right now (e.g., upcoming competition), whether they have a specific barrier preventing their training (e.g., injury), and if they have a coach (these latter variables were not analyzed). Then, they completed the following questionnaires: 1) DARQ; 2) BIS/BAS; 3) SWLS; 4) Exercise Commitment Scale [34]; and 5) a modified version of the International Physical Activity Questionnaire [36].

The first three questionnaires were described in Study 1. The Exercise Commitment Scale [33] was used to assess more proximal predictors of exercise, to test whether the variables assessed by this scale mediate the relationship between dissonance variables and exercise. This scale consists of the following subscales: 1) want to commitment (3 items; e.g., “I am determined”; Cronbach’s alpha = .94); 2) have to commitment (3 items; e.g., “I feel obligated to exercise”; Cronbach’s alpha = .72); 3) satisfaction (3 items; e.g., “Exercise is satisfying”; Cronbach’s alpha = .90); 4) social constraints (3 items; e.g., “Keep exercising to please others”; Cronbach’s alpha = .75); 5) personal investment (3 items; e.g., “Invested a lot of effort”; Cronbach’s alpha = .75); 6) involvement alternatives (4 items; e.g., “Other things are more fun”; Cronbach’s alpha = .89); and 7) social support (3 items; e.g., “People support my exercising”; Cronbach’s alpha = .80). Responses to each item were made on a 1 (strongly disagree) to 7 (strongly agree) scale.

A modified version of the International Physical Activity Questionnaire [36] was used to measure self-reported vigorous and moderate exercise over the last 7 days as well as the average time on one day. The instructions were as follows:

Think about the time you spent being physically active in the last 7 days. Think about the activities you do at work, as part of your house and yard work, to get from place to place, and in your spare time for recreation, exercise, or sport. Now, think about all the VIGOROUS activities which take HARD PHYSICAL EFFORT that you did in the last 7 days. Vigorous activities make you breathe much harder than normal and may include heavy lifting, digging, aerobics, or fast bicycling. Think only about those physical activities that you did for at least 10 minutes at a time.

Participants were then asked, “During the LAST 7 DAYS, on how many days did you do VIGOROUS physical activities?” Responses could range from 0 to 7. Then, they were asked, “How much time did you usually spend doing VIGOROUS physical activities on one of those days? That is, what is the average amount of time you spent each day over the last 7 days doing VIGOROUS physical activities?” Responses could range from 0 to 180 min.

Next, participants were asked about moderate exercise as follows:

Now, think about activities which take MODERATE PHYSICAL EFFORT that you did in the last 7 days. Moderate physical activities make you breathe somewhat harder than normal and may include carrying light loads, bicycling at a regular pace, or doubles tennis. Do not include walking. Again, think about only those physical activities that you did for at least 10 minutes at a time.

Then, participants were asked the same questions as above about number of days and amount of time for moderate exercise. They were also asked about walking; these variables were not analyzed.

We did not calculate one value for overall metabolic equivalent value (which multiplies time by different values depending on exercise intensity) for exercise because some participants indicated at the end of the study that they misunderstand the time measure. Because it was impossible to know which participants misunderstood these instructions (i.e., some participants may have misunderstood but not indicated such in the comments because they were not aware of their misunderstanding), we analyzed the results for time and days separately.

### Results

Participants reported a wide range of exercises as their primary exercise: cycling (*n* = 14); running (*n* = 71); walking (*n* = 65); weight lifting (*n* = 81); yoga (*n* = 12); swimming (*n* = 3); aerobics/cardio (*n* = 24); unable to categorize (*n* = 8). Unlike Study 1, most participants were not cyclists; instead, most of Study 2’s participants were weightlifters, and cycling was the fifth most popular exercise.

As shown in Table 6, dissonance arousal was not negatively correlated with vigorous or moderate days. In addition, dissonance arousal was positively correlated with vigorous and moderate time. These correlations were inconsistent with predictions. On the other hand, dissonance reduction was correlated positively with vigorous and moderate days and time. These correlations were consistent with predictions. Finally, men engaged in more vigorous and moderate exercise than did women.

**Table 6:** Correlations of exercise variables with dissonance-related variables, age, and gender (Study 2).

	Vig Days	Vig Time	Mod Days	Mod Time
**Ds Aro**	-.01	.18***	-.07	.22***
**Eff Aro**	.08	.27***	-.03	.28***
**Dec Aro**	-.00	.18***	-.05	.23***
**Ind Aro**	-.09	.02	-.10	.07
**Ds Red**	.19***	.12**	.20***	.13**
**Eff Red**	.21***	.13**	.18***	.13**
**Dec Red**	.14**	.01	.15	-.00
**Ind Red**	.11*	.15**	.15**	.19***
**Age**	-.07	-.08	-.04	-.07
**Gender**	-.19***	-.26***	-.10*	-.13**

Note.

* is *p* < .10

** is *p* < .05

*** is *p* < .01 (two-tailed tests; because some predictions were directional, we used the *p* < .10 as a cutoff value, which would be *p* < .05 one-tailed). Gender was coded 1 = male and 2 = female, so higher scores on gender are female. Vig Days = number of days exercised vigorously; Vig Time = number of minutes exercised vigorously; Mod Days = number of days exercised moderately; Mod Time = number of minutes exercised moderately; Ds Aro = dissonance arousal overall; Eff Aro = effort justification arousal; Dec Aro = decision arousal; Ind Aro = induced compliance arousal; Ds Red = dissonance reduction overall; Eff Red = effort justification reduction; Dec Red = decision reduction; Ind Red = induced compliance reduction.

Because gender and age correlated with exercise variables in Study 1 and they tended to correlate with exercise variables in Study 2, we computed partial correlations between exercise and dissonance variables controlling for gender and age. As shown in Table 7, these statistical controls did not alter the pattern of results observed in the bivariate correlations.

**Table 7:** Partial correlations of exercise variables with dissonance variables controlling for gender and age (Study 2).

	Vig Days	Vig Time	Mod Days	Mod Time
**Dis Aro**	-.01	.18***	-.08	.22***
**Effort Aro**	.07	.26***	-.04	.27***
**Dec Aro**	.00	.19***	-.05	.23***
**Ind Aro**	-.10*	.02	-.10*	.06
**Dis Red**	.18***	.10*	.19***	.12**
**Effort Red**	.19***	.10	.17***	.11*
**Dec Red**	.15**	.01	.16***	.00
**Ind Red**	.10*	.15**	.15**	.18***

*Note*.

* *p* < .10

** *p* < .05

***p < .01.

Ds Aro = dissonance arousal overall; Eff Aro = effort justification arousal; Dec Aro = decision arousal; Ind Aro = induced compliance arousal; Ds Red = dissonance reduction overall; Eff Red = effort justification reduction; Dec Red = decision reduction; Ind Red = induced compliance reduction.

We next examined correlations of dissonance variables with BIS/BAS, SWLS, age, and gender. As shown in Table 8, dissonance arousal was positively correlated with BIS, but negatively correlated with BAS-Drive, BAS-Reward Responsiveness, Satisfaction with Life, and age. Dissonance arousal was positively correlated with BAS-FS. In contrast, dissonance reduction negatively correlated with BIS, but positively correlated with all BAS subscales and Satisfaction with Life. For the most part, dissonance reduction was not correlated with age and gender, except in two cases.

**Table 8:** Correlations of dissonance variables with BIS, BAS, SWLS, age, and gender (Study 2).

	BIS	BAS-D	BAS-FS	BAS-RR	SWLS	Age	Gender
**Ds Aro**	.58***	-.17***	.13**	-.13**	-.26***	-.24***	.02
**Eff Aro**	.25***	-.14**	.15***	-.20***	-.10*	-.18***	-.04
**Dec Aro**	.55***	-.20***	.14**	-.15**	-.24***	-.17***	.05
**Ind Aro**	.67***	-.10*	.05	.01	-.32***	-.25***	.04
**Ds Red**	-.30***	.59***	.31***	.56***	.51***	.07	-.09
**Eff Red**	-.14**	.52***	.30***	.55***	.38***	-.01	-.14**
**Dec Red**	-.41***	.47***	.18***	.40***	.51***	.16***	-.03
**Ind Red**	-.23***	.48***	.29***	.43***	.38***	.04	-.04

*Note*.

* *p* < .10

** *p* < .05

***p < .01.

Ds Aro = dissonance arousal overall; Eff Aro = effort justification arousal; Dec Aro = decision arousal; Ind Aro = induced compliance arousal; Ds Red = dissonance reduction overall; Eff Red = effort justification reduction; Dec Red = decision reduction; Ind Red = induced compliance reduction. BIS = Behavioral Inhibition Scale; BAS = Behavioral Activation Scale (D = drive; FS = fun-seeking; RR = reward responsiveness); SWLS = Satisfaction with Life Scale.

Because age and gender correlated with some key variables, the correlations were re-run controlling for age and gender. As shown in Table 9, the pattern of results was similar to those with the bivariate correlations.

**Table 9:** Correlations of BIS/BAS and SWLS with exercise variables, controlling for age and gender (Study 2).

	Vig Days	Vig Time	Mod Days	Mod Time
**BIS**	-.16***	-.10	-.14**	-.07
**BAS D**	.12**	.07	.20***	.16***
**BAS FS**	.18***	.19***	.20***	.20***
**BAS RR**	.04	.00	.19***	.09
**SWLS**	.22***	.21***	.15**	.15**

*Note*.

* *p* < .10

** *p* < .05

***p < .01.

Vig Days = number of days exercised vigorously; Vig Time = number of minutes exercised vigorously; Mod Days = number of days exercised moderately; Mod Time = number of minutes exercised moderately; BIS = Behavioral Inhibition Scale; BAS = Behavioral Activation Scale (D = drive; FS = fun-seeking; RR = reward responsiveness); SWLS = Satisfaction with Life Scale.

Because BIS, BAS, and SWLS were significantly correlated with the exercise variables, we conducted multiple regressions in which dissonance-related variables (that were significantly correlated with exercise variables) and BIS (as well as BAS and SWLS) were used as simultaneous predictors of exercise. We did these analyses to test whether dissonance variables related to exercise because of BIS (or BAS or SWLS). To reduce the number of analyses, we created a BAS composite variable (average of the three BAS subscales) and focused only on dissonance reduction overall and dissonance arousal overall. As shown in Table 10, the results suggest that some correlations of dissonance-related variables with exercise may have been partially due to BAS or SWLS. However, other correlations of dissonance-related variables with exercise suggest that BIS, BAS, and SWLS did not explain them.

**Table 10:** Partial correlations of exercise variables with dissonance variables and BIS, BAS, and SWLS (the latter variables were simultaneous predictors; Study 2).

	Vig Days	Vig Time	Mod Days	Mod Time
**Ds Aro**	.16**	.32***	.03	.34***
**BIS**	-.29***	-.31***	-.14**	-.28***
**Ds Red**	.14**	.08	.16**	.10*
**BIS**	-.15**	-.12**	-.10*	-.06
**Ds Aro**	.00	.19***	-.06	.24***
**BAS**	.15**	.14**	.24***	.21***
**Ds Red**	.12**	.06	.07	.02
**BAS**	.05	.07	.16***	.14**
**Ds Aro**	.05	.24***	-.03	.27***
**SWLS**	.22***	.26***	.13**	.21***
**Ds Red**	.10	.02	.14**	.07
**SWLS**	.14**	.17**	.06	.09

*Note*.

* *p* < .10

** *p* < .05

***p < .01.

Ds Aro = dissonance arousal overall; Ds Red = dissonance reduction overall; BIS = Behavioral Inhibition Scale; BAS = Behavioral Activation Scale overall; SWLS = Satisfaction with Life Scale.

Next, we examined correlations of exercise commitment variables with exercise and dissonance variables. As shown in Table 11, exercise was positively correlated with most exercise commitment subscales, but it was negatively correlated with involvement alternatives. In addition, dissonance arousal was negatively correlated with want-to, satisfaction, and social support. Dissonance arousal was positively correlated with social constraints and involvement alternatives. In contrast, dissonance reduction was positively correlated with want-to, have-to, satisfaction, personal investment, and social support. Dissonance reduction was negatively correlated with involvement alternatives.

**Table 11:** Partial correlations of exercise variables and dissonance variables with commitment to exercise variables, controlling for gender and age (Study 2).

	Vig Days	Vig Time	Mod Days	Mod Time	Dis Aro	Dis Red
**Want to**	.41***	.21***	.37***	.19***	-.20***	.41***
**Have to**	.30***	.14**	.32***	.25***	.08	.30***
**Satisfaction**	.36***	.19***	.33***	.17***	-.16***	.41***
**Soc Constraint**	.13**	.23***	.10*	.33***	.36***	.04
**Pers Invest**	.50***	.31***	.35***	.30***	-.05	.30***
**Inv Alternat**	-.24***	-.02	-.22***	.06	.31***	-.17***
**Soc Support**	.16***	.11*	.24***	.16***	-.12**	.35***

*Note*.

* *p* < .10

** *p* < .05

***p < .01.

Ds Aro = dissonance arousal overall; Ds Red = dissonance reduction overall; Soc Constraint = social constraints; Pers Invest = personal investment; Inv Alternat = involvement alternatives; Soc Support = social support.

#### Mediational analyses

The broad, more distal individual differences of dissonance arousal and dissonance reduction may exert their effects on exercise behavior through more proximal variables of commitment to exercise. Thus, we tested whether commitment to exercise variables mediated the effects of dissonance-related variables (i.e., overall dissonance arousal and dissonance reduction) on exercise. To do this, we used the PROCESS macro [37] in SPSS (Model 4) with 10,000 bootstrap iterations to estimate the indirect effect and its 95% confidence interval. In these analyses, gender and age were included as covariates. These mediational analyses focused on the above correlational results that were significant and suggested possible mediation. For example, both dissonance reduction and want to commit correlated positively with each other, and they both correlated positively with vigorous exercise. Testing the indirect effect and its 95% would assess whether want to commit mediated the effect of dissonance reduction on vigorous exercise. In addition, if correlational results did not suggest possible mediation, then mediation was not tested. For example, because dissonance arousal was not significantly correlated with vigorous days or moderate days, mediational analyses were not conducted on these relationships. We examined each possible mediator separately and did not test parallel multiple mediation because we not interested in assessing how each mediator performed while controlling for other mediators. That is, we suspect that a given individual may use multiple means of reducing exercise-related dissonance over time.

For the relationship between dissonance reduction and vigorous days, the following variables were significant mediators as identified by the 95% confidence intervals of their indirect effects: want to commit; have to commit; personal investment; exercise satisfaction; and involvement alternatives. See Table 12.

**Table 12:** Mediational analyses for dissonance reduction and days of exercise (Study 2).

Dissonance reduction predicted…
**Vigorous Days with Want to Commit as Mediator**
Direct effect: *b* = .03, *SE* = .16, *95% CI* [-.28, .34]
Indirect effect: *b* = .44, *SE* = .09, *95% CI* [.28, .64]*
**Moderate Days with Want to Commit as Mediator**
Direct effect: *b* = .12, *SE* = .16, *95% CI* [-.20, .43]
Indirect effect: *b* = .38, *SE* = .09, *95% CI* [.23, .57]*
**Vigorous Days with Have to Commit as Mediator**
Direct effect: *b* = .25, *SE* = .16, *95% CI* [-.06, .56]
Indirect effect: *b* = .22, *SE* = .07, *95% CI* [.10, .37]*
**Moderate Days with Have to Commit as Mediator**
Direct effect: *b* = .23, *SE* = .16, *95% CI* [-.03, .58]
Indirect effect: *b* = .23, *SE* = .07, *95% CI* [.10, .38]*
**Vigorous Days with Exercise Satisfaction as Mediator**
Direct effect: *b* = .09, *SE* = .16, *95% CI* [-.22, .41]
Indirect effect: *b* = .38, *SE* = .08, *95% CI* [.23, .56]*
**Moderate Days with Exercise Satisfaction as Mediator**
Direct effect: *b* = .17, *SE* = .16, *95% CI* [-.15, .49]
Indirect effect: *b* = .33, *SE* = .09, *95% CI* [.18, .52]*
**Vigorous Days with Personal Investment as Mediator**
Direct effect: *b* = .08, *SE* = .14, *95% CI* [-.20, .37]
Indirect effect: *b* = .38, *SE* = .10, *95% CI* [.21, .60]*
**Moderate Days with Personal Investment as Mediator**
Direct effect: *b* = .25, *SE* = .15, *95% CI* [-.05, .55]
Indirect effect: *b* = .25, *SE* = .07, *95% CI* [.12, .41]*
**Vigorous Days with Involvement Alternatives as Mediator**
Direct effect: *b* = .37, *SE* = .15, *95% CI* [.07, .67]
Indirect effect: *b* = .10, *SE* = .04, *95% CI* [.02, .20]*
**Moderate Days with Involvement Alternatives as Mediator**
Direct effect: *b* = .41, *SE* = .15, *95% CI* [.11, .71]
Indirect effect: *b* = .09, *SE* = .05, *95% CI* [.01, .19]*
**Vigorous Days with Social Support as Mediator**
Direct effect: *b* = .39, *SE* = .17, *95% CI* [.07, .71]
Indirect effect: *b* = .11, *SE* = .07, *95% CI* [-.04, .25]
**Moderate Days with Social Support as Mediator**
Direct effect: *b* = .33, *SE* = .16, *95% CI* [.01, .65]
Indirect effect: *b* = .18, *SE* = .08, *95% CI* [.03, .33]*
**Social Constraints**
Dis Reduction did not relate to Social Constraints, so mediation was not tested

*Note*. Significant mediators, as identified by the 95% confidence intervals of their indirect effects, are indicated with an asterisk (*).

### Discussion

The results from Study 2 conceptually replicated the results from Study 1 by demonstrating that dissonance reduction correlated positively with exercise as measured by number of vigorous and moderate days. However, dissonance arousal, for the most part, was not significantly correlated with number of days (however, induced arousal did correlate negatively with number of days).

The results with self-reported time, however, were not as clear cut. Dissonance reduction correlated positively with self-reported time, as predicted. However, dissonance arousal also correlated positively with time; this is opposite to predictions if self-reported exercise time is a measure of effectively enacting one’s commitments to an exercise goal.

We suspect that these correlations with time may be problematic. In open-ended comments and emails, some participants informed us that they misunderstood the instructions for reporting time, and instead of reporting time for an average day, they reported time for the entire week.

## Study 3

Because it is impossible to know how widespread the misunderstanding of the instructions for reporting time was, we thought it important to conduct another study. Thus, in Study 3, we changed the instructions for the time measure to make them clearer about average day (not weekly time), to see if the results from Study 2 would replicate.

### Method

#### Participants

Recruited from Prolific to complete a study on physical exercise and personality, participants were 292 individuals (116 male, 176 female) who lived in the USA, UK, or Australia. Participants were of the following ethnicities: 211 European/White, 18 African/Black, 37 Asian, 14 Hispanic/Latino, and 12 other. Participants were between 18 and 74 years of age (*M* = 31.55, *SD* = 12.23). An additional 11 respondents completed the survey but their data was not analyzed because they did not correctly answer attention check questions embedded in the questionnaires or because they did not give sensible answers to open-ended questions. Participants were paid $3.30 USD for their time.

#### Procedure

In addition to switching from Mturk to Prolific, the other differences between this study and Study 2 were: 1) the instructions for reporting time exercised were modified to make them clearer; and 2) all exercise-related questions were administered after the DARQ and other personality questionnaires. The new time instructions were: “What is the AVERAGE amount of time you spent each DAY over the last 7 days doing MODERATE physical activities? Don’t give the time for the entire week; just give the time for a typical day.”

### Results

Participants reported a wide range of exercises as their primary exercise: cycling (*n* = 20); running (*n* = 56); walking (*n* = 97); weight lifting (*n* = 61); yoga (*n* = 8); swimming (*n* = 5); aerobics/cardio (*n* = 23); unable to easily place into one of the larger categories (*n* = 22). Unlike Study 1, most participants were not cyclists; instead, most of Study 3’s participants were walkers, and cycling was the fifth most popular exercise.

As shown in Table 13, dissonance arousal correlated negatively with number of exercise days; these correlations were significant for vigorous days but not quite significant for moderate days. In contrast, dissonance reduction correlated positively with number of exercise days. Correlations with exercise time were for the most part non-significant, but in predicted directions. Gender also correlated with vigorous exercise days and time. This correlation indicated that women engaged in less vigorous exercise.

**Table 13:** Correlations of exercise variables with dissonance-related variables, age, and gender (Study 3).

	Vig Days	Vig Time	Mod Days	Mod Time
**Ds Aro**	-.14**	-.06	-.08	-.06
**Eff Aro**	-.14**	-.11*	-.11*	-.07
**Dec Aro**	-.13**	-.04	-.06	-.04
**Ind Aro**	-.08	-.01	-.04	-.05
**Ds Red**	.16***	.12**	.12**	.07
**Eff Red**	.19***	.17***	.14**	.08
**Dec Red**	.10*	.09	.10*	.05
**Ind Red**	.06	.02	.03	.01
**Age**	-.07	-.07	-.07	-.08
**Gender**	-.21***	-.21***	-.07	-.05

*Note*.

* *p* < .10

** *p* < .05

***p < .01.

Ds Aro = dissonance arousal overall; Eff Aro = effort justification arousal; Dec Aro = decision arousal; Ind Aro = induced compliance arousal; Ds Red = dissonance reduction overall; Eff Red = effort justification reduction; Dec Red = decision reduction; Ind Red = induced compliance reduction.

The bivariate correlations between dissonance-related variables and exercise remained significant when statistically controlling for gender and age, as shown in Table 14. The results with number of days exercised replicate the results from Study 2. However, the results with number of minutes (time) do not. Whereas Study 2 found unexpected positive correlations between dissonance arousal and time, Study 3 found negative correlations, as predicted.

**Table 14:** Partial correlations of exercise variables with dissonance variables controlling for gender and age (Study 3).

	Vig days	Vig time	Mod days	Mod time
**Ds Aro**	-.15***	-.06	-.10*	-.09
**Eff Aro**	-.17***	-.13**	-.13**	-.09
**Dec Aro**	-.13**	-.03	-.07	-.05
**Ind Aro**	-.08	-.00	-.05	-.07
**Ds Red**	.17***	.14**	.13**	.08
**Eff Red**	.19***	.16***	.14**	.08
**Dec Red**	.12**	.11*	.12**	.07
**Ind Red**	.08	.03	.04	.03

*Note*.

* *p* < .10

** *p* < .05

***p < .01.

Ds Aro = dissonance arousal overall; Eff Aro = effort justification arousal; Dec Aro = decision arousal; Ind Aro = induced compliance arousal; Ds Red = dissonance reduction overall; Eff Red = effort justification reduction; Dec Red = decision reduction; Ind Red = induced compliance reduction.

In Table 15, we present correlations of dissonance variables with BIS, BAS, SWLS, age, and gender. Replicating results from previous studies, dissonance arousal was positively correlated with BIS and negatively correlated with some BAS subscales as well as SWLS and age. Women also tended to score higher on dissonance arousal than men did. In contrast, dissonance reduction was negatively correlated with BIS and positively correlated with some BAS as well as SWLS and age. Women and men did not differ significantly on dissonance reduction.

**Table 15:** Correlations of dissonance variables with BIS, BAS, SWLS, age, and gender (Study 3).

	BIS	BAS-D	BAS-FS	BAS-RR	SWLS	Age	Gender
**Ds Aro**	.63***	-.23***	.04	-.05	-.42***	-.32***	.14**
**Eff Aro**	.33***	-.20***	.06	-.21***	-.38***	-.29***	.05
**Dec Aro**	.54***	-.22***	.07	-.05	-.41***	-.28***	.14**
**Ind Aro**	.67***	-.16***	-.02	.11*	-.27***	-.23***	.14**
**Ds Red**	-.32***	.46***	.23***	.32***	.41***	.16**	-.03
**Eff Red**	-.11*	.37***	.19***	.31***	.29***	.07	-.08
**Dec Red**	-.34***	.34***	.14**	.28***	.42***	.19***	-.01
**Ind Red**	-.30***	.34***	.21***	.13**	.22***	.13**	.01

*Note*.

* *p* < .10

** *p* < .05

***p < .01.

Ds Aro = dissonance arousal overall; Eff Aro = effort justification arousal; Dec Aro = decision arousal; Ind Aro = induced compliance arousal; Ds Red = dissonance reduction overall; Eff Red = effort justification reduction; Dec Red = decision reduction; Ind Red = induced compliance reduction.

Next, we computed correlations of exercise variables with BIS, BAS, and SWLS. As shown in Table 16, BIS was not significantly correlated with exercise. However, BAS-Drive was positively correlated with exercise, whereas BAS-Fun-Seeking and BAS-Reward-Responsiveness were not significantly correlated with exercise. Satisfaction with Life was correlated positively with exercise.

**Table 16:** Correlations of BIS/BAS and SWLS with exercise variables (Study 3).

	Vig Days	Vig Time	Mod Days	Mod Time
**BIS**	-.08	-.08	.00	-.07
**BAS D**	.19***	.14**	.16***	.15**
**BAS FS**	-.06	.05	.11*	.09
**BAS RR**	.04	.07	.07	.10
**SWLS**	.17***	.18***	.16***	.13**

*Note*.

* *p* < .10

** *p* < .05

***p < .01.

Next, we conducted the same multiple regressions used in Study 2 to test whether dissonance variables related to exercise because of BIS (or BAS or SWLS). To reduce the number of analyses, we created a BAS composite variable (average of the three BAS subscales) and focused only on dissonance reduction overall and dissonance arousal overall. As shown in Table 17, the results suggest that some correlations of dissonance-related variables with exercise may have been partially due to BAS or SWLS. However, other correlations of dissonance-related variables with exercise suggest that BIS, BAS, and SWLS did not explain them.

**Table 17:** Partial correlations of exercise variables with dissonance variables and BIS, BAS, and SWLS (the latter variables were simultaneous predictors; Study 3).

	Vig Days	Vig Time	Mod Days	Mod Time
**Ds Aro**	-.15**	-.02	-.11*	-.03
**BIS**	.02	-.05	.07	-.04
**Ds Red**	.14**	.10*	.13**	.05
**BIS**	-.02	-.04	.05	-.05
**Ds Aro**	-.13**	-.05	-.06	-.05
**BAS**	.07	.11*	.14**	.13**
**Ds Red**	.14**	.08	.06	.01
**BAS**	.02	.07	.11*	.12**
**Ds Aro**	-.08	.02	-.01	-.00
**SWLS**	.12**	.17***	.14**	.12**
**Ds Red**	.10*	.06	.06	.01
**SWLS**	.12**	.14**	.12**	.12**

*Note*.

* *p* < .10

** *p* < .05

***p < .01.

As in Study 2, we next examined correlations of exercise commitment variables with exercise and dissonance variables. As shown in Table 18, exercise was positively correlated with most exercise commitment subscales, but it was negatively correlated with involvement alternatives and not significantly correlated with social constraints. In addition, dissonance arousal was negatively correlated with want-to, satisfaction, personal investment, and social support. Dissonance arousal was positively correlated with social constraints and involvement alternatives. In contrast, dissonance reduction was positively correlated with want-to, have-to, satisfaction, personal investment, and social support. Dissonance reduction was negatively correlated with involvement alternatives.

**Table 18:** Partial correlations of exercise variables and dissonance variables with commitment to exercise variables, controlling for gender and age (Study 3).

	Vig Days	Vig Time	Mod Days	Mod Time	Dis Aro	Dis Red
**Want to**	.54***	.29***	.41***	.21***	-.21***	.24***
**Have to**	.34***	.19***	.33***	.14**	.05	.09
**Satisfaction**	.39***	.25***	.28***	.14**	-.21***	.32***
**Soc Constraints**	.02	.08	-.01	.08	.23***	-.05
**Pers Investment**	.49***	.30***	.35***	.21***	-.18***	.20***
**Inv Alternat**	-.34***	-.20***	-.21***	-.08	.18***	-.11*
**Soc Support**	.24***	.11*	.20***	.11*	-.15**	.28***

*Note*.

* *p* < .10

** *p* < .05

***p < .01.

#### Mediational analyses

As in Study 2, mediational analyses were conducted to test whether commitment to exercise variables mediated the effects of dissonance-related variables (i.e., overall dissonance arousal and dissonance reduction) on exercise. The methods to test mediation were identical to those used in Study 2. Also, the mediational analyses focused on the above correlational results that were significant and suggested possible mediation. Because dissonance arousal was only weakly related to moderate days, mediations for the dissonance arousal and moderate days relationships were not tested.

For the relationship between dissonance reduction and vigorous days (and moderate days), the following variables were significant mediators as identified by the 95% confidence intervals of their indirect effects: want to commit; exercise satisfaction; personal investment; and social support. See Table 19.

**Table 19:** Mediational analyses for dissonance reduction and days of exercise (Study 3).

Dissonance Reduction Predicted …
**Vigorous Days with Want to Commit as Mediator**
Direct effect: *b* = .13, *SE* = .15, *95% CI* [-.17, .43]
Indirect effect: *b* = .38, *SE* = .10, *95% CI* [.19, .57]*
**Moderate Days with Want to Commit as Mediator**
Direct effect: *b* = .12, *SE* = .17, *95% CI* [-.23, .46]
Indirect effect: *b* = .30, *SE* = .08, *95% CI* [.15, .47]*
**Vigorous Days with Have to Commit as Mediator**
Dis Reduction did not relate, so mediation was not tested
**Moderate Days with Have to Commit as Mediator**
Dis Reduction did not relate to Diss Reduction, so mediation was not tested
**Vigorous Days with Exercise Satisfaction as Mediator**
Direct effect: *b* = .15, *SE* = .17, *95% CI* [-.18, .48]
Indirect effect: *b* = .35, *SE* = .08, *95% CI* [.21, .51]*
**Moderate Days with Exercise Satisfaction as Mediator**
Direct effect: *b* = .15, *SE* = .19, *95% CI* [-.21, .52]
Indirect effect: *b* = .26, *SE* = .07, *95% CI* [.13, .41]*
**Vigorous Days with Personal Investment as Mediator**
Direct effect: *b* = .22, *SE* = .15, *95% CI* [-.08, .52]
Indirect effect: *b* = .29, *SE* = .09, *95% CI* [.12, .48]*
**Moderate Days with Personal Investment as Mediator**
Direct effect: *b* = .20, *SE* = .18, *95% CI* [-.15, .55]
Indirect effect: *b* = .22, *SE* = .07, *95% CI* [.09, .37]*
**Vigorous Days with Involvement Alternatives as Mediator**
Direct effect: *b* = .40, *SE* = .16, *95% CI* [.08, .72]
Indirect effect: *b* = .11, *SE* = .06, *95% CI* [-.00, .23]
**Moderate Days with Involvement Alternatives as Mediator**
Direct effect: *b* = .35, *SE* = .18, *95% CI* [-.01, .70]
Indirect effect: *b* = .07, *SE* = .05, *95% CI* [-.00, .17]
**Vigorous Days with Social Support as Mediator**
Direct effect: *b* = .34, *SE* = .17, *95% CI* [-.01, .68]
Indirect effect: *b* = .17, *SE* = .06, *95% CI* [.07, .30]*
**Moderate Days with Social Support as Mediator**
Direct effect: *b* = .26, *SE* = .19, *95% CI* [-.11, .63]
Indirect effect: *b* = .16, *SE* = .06, *95% CI* [.05, .30]*
**Social Constraints**
Dis Reduction did not relate to Social Constraints, so mediation was not tested, for either vigorous or moderate days of exercise

*Note*. Significant mediators, as identified by the 95% confidence intervals of their indirect effects, are indicated with an asterisk (*).

Unlike the results of Study 2, in Study 3, dissonance arousal correlated with number of days of vigorous exercise, and thus mediation was tested using the same methods as in Study 2. For the relationship between dissonance arousal and vigorous days, the following variables were significant mediators as identified by the 95% confidence intervals of their indirect effects: want to commit; exercise satisfaction; personal investment; involvement alternatives; and social support. However, it is important to note that all of these mediators were negatively mediators. For example, dissonance arousal was negatively correlated with want to commit, but want to commit was positively correlated with vigorous exercise. See Table 20.

**Table 20:** Mediational analyses for dissonance arousal and days of exercise (Study 3).

Dissonance Arousal Predicted…
**Vigorous Days with Want to Commit as Mediator**
Direct effect: *b* = -.08, *SE* = .10, *95% CI* [-.28, .13]
Indirect effect: *b* = -.23, *SE* = .06, *95% CI* [-.36, -.12]–negative mediator
**Vigorous Days with Have to Commit as Mediator**
Dis Arousal did not relate, so mediation was not tested
**Vigorous Days with Exercise Satisfaction as Mediator**
Direct effect: *b* = -.15, *SE* = .11, *95% CI* [-.38, .07]
Indirect effect: *b* = -.16, *SE* = .05, *95% CI* [-.27, -.07]–negative mediator
**Vigorous Days with Personal Investment as Mediator**
Direct effect: *b* = -.14, *SE* = .11, *95% CI* [-.35, .07]
Indirect effect: *b* = -.18, *SE* = .07, *95% CI* [-.32, -.06]–negative mediator
**Vigorous Days with Involvement Alternatives as Mediator**
Direct effect: *b* = -.19, *SE* = .12, *95% CI* [-.42, .04]
Indirect effect: *b* = -.12, *SE* = .05, *95% CI* [-.22, -.04]–negative mediator
**Vigorous Days with Social Support as Mediator**
Direct effect: *b* = -.25, *SE* = .12, *95% CI* [-.48, -.01]
Indirect effect: *b* = -.07, *SE* = .03, *95% CI* [-.14, -.01]–negative mediator
**Social Constraints**
Dis Arousal did not relate to Social Constraints, so mediation was not tested

*Note*. Significant mediators, as identified by the 95% confidence intervals of their indirect effects, are indicated with an asterisk (*).

## General discussion

The present results suggested that individual differences in dissonance arousal relate to less exercise and that individual differences in dissonance reduction relate to more exercise. This pattern of results was generally consistent across the three studies that had three different sources of participants. Moreover, these results occurred when amount of exercise was measured using annual GPS data from an online platform and when it was measured using self-reports of the number of days exercised in the last week.

One limitation to the studies is that the predicted and obtained correlation coefficients were in the range of what some might label as small. However, as noted by Funder and Ozer [38], effect sizes need to be interpreted with regard to a frame of reference. The measures of individual differences in dissonance arousal and reduction refer to responses to general dissonance-related situations (i.e., decisions, effort, and counterattitudinal behaviors). The fact that these general individual differences measures that mention nothing about exercise relate to exercise behavior is, in our opinion, impressive. In addition, the current effect sizes may not be all that unusual. For instance, a project reviewing 708 correlations derived from meta-analyses of social and personality psychology research revealed that the average effect r was .19 [39]. As Funder and Ozer [38, p. 166] wrote, “Smaller effect sizes are not merely worth taking seriously. They are also more believable.”

Some inconsistencies with these general patterns of results occurred. For example, in Study 1, the predicted pattern of results occurred primarily for cycling, but not for running. We suspect that our Study 1 sample may have included some unusual runners, because Studies 2 and 3 included a broad range of exercises and the results were consistent with predictions (examination of only runners from Studies 2 and 3 were also consistent with predictions). We suspect that perhaps some oddities about the runners in Study 1 caused the null effects. In Study 2, self-reported time spent exercising on an average day during the week was positively correlated with dissonance arousal. We suspected this latter correlation may have resulted because some participants misunderstood the instructions. When the instructions were changed to be clearer in Study 3, this unexpected correlation did not occur. Another possibility is that perhaps time is not the best measure of the construct of “effective behavior”, and time exercising is influenced by many variables unrelated to dissonance processes. Number of days appears to be a better measure of effective behavior.

Statistically controlling for BIS/BAS and satisfaction with life did not appreciably alter the correlations between the dissonance-related variables and exercise. These results suggest that even though BIS/BAS and satisfaction with life correlate with exercise, they likely do not explain why dissonance-related variables correlate with exercise. Thus, these results suggest that these “third variables” do not fully explain why dissonance-related variables correlated with exercise.

Studies 2 and 3 also provided evidence suggesting possible mediators of the relationship between dissonance-related variables and exercise. In particular, the relationship between dissonance reduction and number of days exercised was mediated by want to commit, personal investment, exercise satisfaction, and involvement alternatives in both studies. Dissonance arousal had significant negative pathways on days of exercise through want to commit, satisfaction, personal investment, involvement alternatives, and social support. This suggests that greater dissonance arousal was associated with reduced exercise to the extent that it increased involvement alternatives and decreased want to, satisfaction, personal investment, and social support.

Another limitation is that the present studies all contained correlational results so the direction of causality is unknown. Our theoretical prediction was that dissonance-related variables influence exercise behavior. However, it is also possible that exercise behavior influences individual differences in dissonance-related variables. We suspect that both directions of causality may occur. Third variables may also explain the correlations. We tried to address some possible third variables (e.g., BIS/BAS, SWLS, age, gender), and did not find that these third variables completely explained the observed relationships. Future research using longitudinal designs could be used to test whether dissonance-related variables causally influence exercise levels. Other future research could examine how various cognitive processes involved in dissonance reduction specifically predict engaging in physical exercise over time [40].

Several previous revisions of dissonance theory cast dissonance reduction as an irrational and maladaptive psychological process. The present results and the action-based model of dissonance, however, suggest otherwise. Dissonance reduction was associated with exercising more often, which some might regard as an adaptive behavior. This may be especially the case in this current society in which the lack of physical exercise contributes to many physical health conditions (e.g., cardiovascular disease, high blood pressure, Type 2 diabetes) and exercising contributes to many health benefits (e.g., greater life satisfaction, less depression, less inflammation, improved brain function) [41–43].

In conclusion, the present research suggests that individuals who scored higher in dissonance reduction (as measured by a self-report questionnaire) engaged in exercise more often, whereas individuals who scored higher in dissonance arousal engaged in exercise less often. These results occurred even when the “third variables” of behavioral inhibition and activation as well as satisfaction with life were statistically controlled, suggesting that the dissonance-related variables are associated with acting effectively with regard to engaging in physical exercise more often.

## Supporting information

S1 File (RAR)
